# Hesitation and Refusal Factors in Individuals' Decision-Making Processes Regarding a Coronavirus Disease 2019 Vaccination

**DOI:** 10.3389/fpubh.2021.626852

**Published:** 2021-04-21

**Authors:** Arcadio A. Cerda, Leidy Y. García

**Affiliations:** Faculty of Economics and Business, University of Talca, Talca, Chile

**Keywords:** perceived benefit, health promotion, treatment refusal, health policy, vaccine, Chile

## Abstract

**Introduction:** Considering the global prevalence of coronavirus disease 2019 (COVID-19), a vaccine is being developed to control the disease as a complementary solution to hygiene measures—and better, in social terms, than social distancing. Given that a vaccine will eventually be produced, information will be needed to support a potential campaign to promote vaccination.

**Objective:** The aim of this study was to determine the variables affecting the likelihood of refusal and indecision toward a vaccine against COVID-19 and to determine the acceptance of the vaccine for different scenarios of effectiveness and side effects.

**Materials and Methods:** A multinomial logistic regression method based on the Health Belief Model was used to estimate the current methodology, using data obtained by an online anonymous survey of 370 respondents in Chile.

**Results:** The results indicate that 49% of respondents were willing to be vaccinated, with 28% undecided or 77% of individuals who would potentially be willing to be inoculated. The main variables that explained the probability of rejection or indecision were associated with the severity of COVID-19, such as, the side effects and effectiveness of the vaccine; perceived benefits, including immunity, decreased fear of contagion, and the protection of oneself and the environment; action signals, such as, responses from ones' family and the government, available information, and specialists' recommendations; and susceptibility, including the contagion rate per 1,000 inhabitants and relatives with COVID-19, among others. Our analysis of hypothetical vaccine scenarios revealed that individuals preferred less risky vaccines in terms of fewer side effects, rather than effectiveness. Additionally, the variables that explained the indecision toward or rejection of a potential COVID-19 vaccine could be used in designing public health policies.

**Conclusions:** We discovered that it is necessary to formulate specific, differentiated vaccination-promotion strategies for the anti-vaccine and undecided groups based on the factors that explain the probability of individuals refusing or expressing hesitation toward vaccination.

## Introduction

The pandemic—derived from the coronavirus disease 2019 (hereafter, “COVID-19”) and characterized by a severe acute respiratory syndrome coronavirus 2 (SARS-CoV-2) infection—has had global effects. Furthermore, it has impacted people's lives, physical and mental health, and economic situation (1–3). Studies indicated that individuals were willing to pay up to $290 for a COVID-19 vaccine, while 10 to 20% would refuse to pay for a vaccine altogether (4, 5). Some governments, such as Australia's, already have announced that a COVID-19 vaccine will be available at no cost, while other countries anticipate that it will be affordable by a majority of people; despite this, uncertainty still exists regarding its effectiveness and side effects in the medium and long term.

Currently, there are several vaccines against COVID-19 that can be manufactured and marketed. During December 2020, several obtained emergency approvals from different health agencies, for example, Moderna in the United States, Pfizer-Biontech in the United States and in Europe, Osford-AstraZeneca in the United Kingdom, and Sinopharm in China. Other vaccines, such as, Sputnik V from Russia, are in phase III and have not yet received approval from the European Medicines Agency (EMA). However, once the vaccine is available, it is important to determine the motivations and health beliefs that will contribute to the decision to be vaccinated and herd immunity can be achieved. By knowing the health beliefs that promote vaccination acceptance, appropriate target campaigns that promote vaccination can be formulated.

According to Jones et al. (6), messages will generate optimal behavioral changes if they affect perceived barriers, benefits, self-efficacy, and threats to achieve broader vaccine acceptance. This should be considered by different governments to implement a vaccination program to combat COVID-19 (7), because, as indicated by Henderson et al. (8) and Ward et al. (9), trust in public health measures and governments influences the willingness to adopt preventive measures. Further, special consideration must be given not only to the anti-vaccine movement and perceptions of a vaccine conspiracy as presented on social media (10, 11) but also to the possible mistrust of institutions or governments regarding vaccinations (12). This is critical when governments attempt to control a pandemic, as a population's hesitancy can soon become a refusal, as mentioned by academics (13). Consequently, this can limit the related public policy's effectiveness, which should be based on knowledge, trust, and legitimacy (12). The roles of social media and physicians in this process could become crucial given their relevance in generating public concerns and influence.

Studies have applied different models trying to explain the willingness-to-pay as well as vaccine acceptance, hesitancy, and refusal to vaccinate, which can vary depending on the context of the individuals and epidemiological conditions of the country. Some researchers have analyzed the variables and factors that explain the probability of getting vaccinated, including individual perceptions and preferences and motivations that affect people's actions (14). Similarly, others have considered that vaccination decisions are also influenced by the individual and group context, and the characteristics of the vaccine (15–17).

As the determinants of vaccine preferences and hesitancy vary across time, place, and vaccines (18), the current situation requires information regarding the determinants that affect people's probability of being vaccinated against COVID-19, as well as the perceived benefits, barriers, threats, and action cues to define the appropriate policies and communication campaign to increase the likelihood of people engaging in health-promoting behavior or, specifically, being vaccinated. In this context, the most appropriate model is the Health Belief Model (HBM). It has been demonstrated that the variable or factor path is not completely defined for this type of model (6). As such, different path relationships can be assumed among variables; that is, the functional form of the HBM is flexible. Therefore, we assumed that there would be a direct relationship between vaccination and the explanatory variables of the HBM. In terms of public policy, the HBM reveals that the variables to be considered relate to perceived barriers, benefits, susceptibility, severity, and cues of actions, among others; in this vein, scarce literature exists regarding the COVID-19 vaccine (5, 19).

Therefore, this study aimed to identify the refusal and hesitancy factors in accepting a hypothetical COVID-19 vaccination in Chile, based on the HBM and using a multinomial logistic regression model (14, 20, 21). This is relevant because the government will need to define the beliefs and variables that should be pursued in communication campaigns to incentivize potential vaccine acceptance (22). This study also provides important information about potential vaccine preferences under three safety and effectiveness scenarios, as well as the main reason to refuse a vaccination. It should be noted that the baseline scenario is the same as the results of the clinical trials (phase III development) of the Pfizer and BioNTech vaccines. Additionally, our study differs from others conducted in the COVID-19 context (5, 19), in that we consider not only the traditional variables from the HBM but also the motivations and cues to action variables (associated with conspiracy theories, the government's communication response, the influence of the family, trusted doctors, and health authorities, which could affect the decision to get vaccinated). By doing so, this study addresses the multiplicity of factors that could influence vaccination decisions (17) and reduces the statistical bias due to the omission of any relevant variables (23).

## Materials and Methods

### Study Design

This was a cross-sectional study. As COVID-19 vaccines will soon be largely available, we framed the study questions around a hypothetical vaccine. First, we evaluated the intention to vaccinate for different effectiveness scenarios and side effects. Second, we identified the determinants of refusal and hesitancy through a multinomial model based on a health beliefs approach similar to previous studies (19, 24). However, we considered complementary explanatory variables that could influence a communication strategy for a vaccination campaign against COVID-19.

### Setting and Period

Given the pandemic and some movement restrictions or quarantines in Chile, this research data were obtained from a self-applied online questionnaire available to respondents 18 years or older through social media, between August 19 and September 13, 2020.

### Sample Size and Recruitment

We reached our population objective by using an online mixed sampling process—including snowball and convenience sampling—but under an active recruitment system. This allowed for an improved, more representative sample population, with a total of 370 respondents, assuming a maximum variance, infinite population, a confidence level of 95%, and a margin error of 5.09, considering the simple random sampling.

### Measurement and Data Collection Techniques

The questionnaire contained four main sections on the COVID-19 situation, beliefs, threats, perception about contracting the illness, and reasons for vaccination, specifically, (a) four questions on susceptibility, three on severity, two on barriers, four on benefits, six on action cues, and two on motivation-related aspects; (b) three questions about the disposition toward vaccination (with 95% regarding effectiveness, 50% regarding effectiveness and minor side effects, and 95% effectivity with unknown side effects), with three possible answers (yes, no, and undecided); (c) a question about the respondent's preferred vaccine developer or producer; (d) reasons for refusal, hesitancy, and dilation to be vaccinated (12 questions); and (e) the respondents' sociodemographic background (eight questions). Most questions were scored on a scale ranging from one (“completely disagree” or “very low” = 1) to five (“completely agree” or “very high” = 5). Additionally, nine questions in section (a) required “yes” or “no” answers, while the questions in sections (b) and (c) were answered as “yes,” “no,” or “hesitancy (undecided)” for each of the previously mentioned alternatives. The scale reliability based on the Cronbach alpha coefficient was 0.757, which is appropriate.

### Ethical Considerations

This study received an exemption status: anonymous and non-sensitive survey research. Before the respondent could access the questionnaire, they were required to give informed consent to participate in the study. They were also informed that the questionnaire was anonymous and voluntary, and respondents' personal information and responses will not be disclosed. Furthermore, they were told the data will be used in aggregated terms.

### Data Analysis

#### Acceptance, Hesitation, and Rejection of a Vaccine Against Coronavirus Disease 2019

We first created scenarios involving three vaccine types, as follows: “Today, would you be willing to receive a free vaccine against COVID-19 that is 95% effective?” (Scenario 1), “Today, would you be willing to receive a free 50% effective vaccine against COVID-19 that will have minor side effects, such as headache, fatigue, muscle aches, pain and rash?” (Scenario 2), and “Today, are you willing to get a free vaccine against COVID-19 with 95% effectiveness, but with unknown side effects?” (Scenario 3). A descriptive statistical analysis was performed of these scenarios, with difference tests on the mean for the different vaccine acceptance rates, and an analysis of the reasons for refusing vaccination.

#### Adapted Health Belief Model

As a theoretical frame of reference, we considered the belief model that has been widely applied to different diseases (5, 19, 24). However, we differed from the available literature by estimating a multinomial model that allowed us to measure the probabilities of individuals' decisions regarding vaccination, remaining undecided, or refusing vaccination entirely. Thus, our estimation method assumes a direct relationship between the variables that make up the HBM factors and the predictor or dependent variable (accepting the vaccine, rejecting it, or expressing indecision). In our case, various factors were considered—or specifically, susceptibility, severity, benefits, barriers, motivations, action cues, and sociodemographic control variables as explanatory variables—to identify the main aspects that influence the decision to vaccinate against COVID-19. For this, the dependent variable (*y*_*i*_) of the result was the following:

$${\text{y}}_{\text{i}}=\{\begin{array}{l} 0\;\text{would}\;\text{be}\;\text{vaccinated}\\ 1\;\text{Would}\;\text{not}\;\text{be}\;\text{vaccinated}\\ 2\;\text{Undecided} \end{array}$$

Specifically, based on Champion and Skinner (25), *perceived benefits* were beliefs in the efficacy of the advised action to reduce the risk or seriousness of the impact of COVID-19, *perceived barriers* were beliefs of the tangible and psychological that limit the decision to get vaccinated, *severity* was opinions of how COVID-19 is considered a serious condition and what its consequences are, *perceived susceptibility* was opinions on the chances of experiencing a risk or getting COVID-19, and *cues to action* were strategies to activate readiness or precipitating forces that make a person feel the need to get vaccinated.

It is highlighted in the literature that studies using the HBM to determine the factors that influence the decision to vaccinate or pay are relatively scarce. Jones et al. (6) considered four factors with 25 variables and five relevant controls of the HBM to evaluate the success of the vaccination campaign against H1N1, while Wong et al. (5) studied five factors with 15 variables and another 10 as control variables to determine the willingness to receive and pay for a vaccine against COVID-19. Both considered perceived benefits, perceived barriers, severity, and perceived susceptibility; and control variables as relevant factors. They differed in that while Jones et al. (6) included self-efficacy, Wong et al. (5) considered cues to action.

It is relevant to understand that signals are the ones that motivate or discourage the action of getting vaccinated. Therefore, we considered six factors, previously defined, with 29 variables including control variables based on previous literature about the HBM. For example, we added other relevant variables such as susceptibility of the infection rate per 1,000 inhabitants, the barrier about anti-vaccine communications on social media, the motivation associated with that the disease was invented by politicians and the pharmaceutical industry, and the cues to action about the government's communication in response and experts recommending the vaccine.

With these variables, we estimated a multinomial logistic regression model in which the dependent variable was categorically unordered with three levels regarding the individual's disposition toward vaccination, defined as “yes,” “no,” or “undecided.” This was estimated under the maximum likelihood estimation method, which is appropriate considering that it does not require the independent variables (which make up the HBM factors) to be statistically independent; that is, it does not contradict the fact that there could be mediating variables, according to what was indicated by Jones et al. (6). The variables were selected using a stepwise statistical procedure and performed using Stata 16 data analysis and statistics software. It should be noted that we report the statistical analysis of the model only for the baseline scenario (Scenario 1: 95% effective vaccine), which provides enough information to formulate public health policies and obtain the best goodness of fit, among the estimates under the three options or individual election (refusal/reject, accept, or hesitancy about the vaccine). Additionally, the model was validated with the analysis of the goodness of fit through the maximum likelihood criterion, Wald's statistic, and multicollinearity test, among others.

Subsequently, the determinants (explanatory variables associated with the HBM factors) of the probability of refusal and hesitancy were analyzed, considering its statistical significance (*p*-value < 0.05). Specifically, the coefficients of the estimation of the multinomial logit model were analyzed; in this, the coefficients (Coef.) were interpreted as the change in one unit in the explanatory variable, and how much variation is generated in the logarithmic probabilities relative to rejecting the vaccine against being vaccinated, given that the variables in the model are held constant. The relative risk ratios (RRR) indicate the expected risk of not getting vaccinated compared with doing so, understanding risk relative to probability (26). These were analyzed in a similar way, but for hesitancy regarding being vaccinated.

## Results

### Data Description

Table 1 presents the general demographic variables under the three vaccine scenarios. The respondents' main demographic data were as follows: 58% were female, 74% had a university degree, 45% had public health care, 5% had no health care, 40% had a relative or friend working in the health-care industry, and 11% were working in health care. The respondents' age categories were homogeneous in three central values, with ~22% representation but extreme, lower values (12 and 17%, the youngest and oldest respondents, respectively). The socioeconomic status of the respondents was primarily middle- and high-income levels, with a frequency of 70% (comparable with the national population of 68%) (27). Additionally, 51% had experienced increased fear of infection in the last 3 months before the survey was conducted.

**Table 1:** Sociodemographic data for three scenarios regarding a hypothetical vaccine against COVID-19.

		**Willingness to vaccinate, 95% effectiveness (baseline)** ***n*** **(%)**			**Willingness to vaccinate, 50% effectiveness; minor side effects** ***n*** **(%)**			**Willingness to vaccinate, 95% effectiveness; unknown side effects** ***n*** **(%)**		
**Variable**	***n* (%)**	**No**	**Undecided**	**Yes**	**No**	**Undecided**	**Yes**	**No**	**Undecided**	**Yes**
**Age**										
18–29	45 (12)	11 (24)	16 (36)	18 (40)	19 (42)	12 (27)	14 (31)	28 (62)	8 (18)	9 (20)
30–39	88 (24)	19 (22)	25 (28)	44 (50)	26 (30)	20 (22)	42 (48)	30 (34)	35 (40)	23 (26)
40–49	82 (22)	24 (29)	20 (25)	38 (46)	40 (49)	15 (18)	27 (33)	45 (55)	17 (21)	20 (24)
50–59	80 (21)	11 (14)	24 (30)	45 (56)	27 (34)	23 (29)	30 (37)	34 (43)	22 (27)	24 (30)
60+	62 (17)	16 (26)	15 (24)	31 (50)	25 (40)	19 (31)	18 (29)	19 (31)	15 (24)	28 (45)
**Gender**										
Female	216 (58)	55 (65)	66 (65)	95 (52)	85 (60)	56 (40)	75 (56)	103 (64)	62 (60)	51 (49)
Male	150 (41)	28 (33)	36 (35)	86 (47)	55 (39)	38 (59)	57 (43)	56 (35)	41 (40)	53 (50)
Not defined	4 (1)	2 (2)	0	2 (1)	1 (1)	1 (1)	2 (1)	3 (2)	0	1 (1)
**Education**										
High school	38 (10)	5 (6)	12 (12)	21 (12)	9 (6)	12 (13)	27 (13)	18 (11)	7 (7)	13 (12)
Technical	57 (15)	15 (18)	18 (18)	24 (13)	25 (18)	17 (18)	15 (11)	29 (18)	16 (15)	12 (11)
University degree	140 (38)	34 (40)	42 (41)	64 (35)	50 (36)	36 (38)	54 (40)	62 (8)	39 (38)	39 (37)
Graduate degree	135 (36)	31 (36)	30 (29)	74 (40)	57 (40)	30 (31)	48 (46)	53 (33)	41 (40)	41 (40)
**Monthly income**										
Less than $569	53 (14)	9 (11)	14 (14)	30 (16)	12 (9)	18 (19)	23 (17)	20 (12)	15 (15)	18 (17)
$570–$953	57 (15)	15 (18)	17 (17)	25 (14)	21 (15)	18 (19)	18 (13)	29 (18)	15 (15)	13 (12)
$954–$1,476	53 (14)	13 (15)	12 (12)	28 (15)	21 (15)	13 (14)	19 (14)	20 (12)	16 (15)	17 (16)
$1,477–$2,186	63 (17)	10 (12)	16 (16)	37 (20)	26 (18)	16 (17)	21 (16)	26 (16)	15 (14)	22 (21)
$2,186+	144 (39)	38 (45)	43 (42)	63 (34)	61 (43)	30 (31)	53 (40)	67 (41)	42 (41)	35 (33)
**Type of health system**										
None	17 (5)	4 (5)	3 (3)	10 (5)	6 (4)	6 (6)	5 (4)	5 (3)	8 (8)	4 (4)
Public	158 (43)	36 (43)	41 (41)	81 (22)	55 (39)	39 (41)	64 (48)	76 (47)	33 (32)	49 (46)
Private	192 (52)	45 (52)	58 (56)	89 (49)	80 (57)	49 (52)	63 (47)	81 (50)	59 (57)	52 (50)
Other	3 (1)	0	0	3 (2)	0	1 (1)	2 (1)	0	3 (3)	0
**Relative work health system**										
No	223 (60)	45 (53)	70 (69)	108 (59)	79 (56)	60 (63)	84 (63)	99 (61)	62 (60)	62 (59)
Yes	147 (40)	40 (47)	32 (31)	75 (41)	62 (44)	35 (37)	50 (37)	63 (39)	41 (40)	43 (41)
**Work health system**										
No	328 (89)	73 (86)	98 (96)	157 (86)	122 (87)	6 (1)	120 (90)	147 (91)	92 (89)	89 (85)
Yes	42 (11)	12 (14)	4 (4)	26 (14)	19 (13)	9 (9)	14 (10)	15 (9)	11 (11)	16 (15)
**Fears of infection have increased in the last 3 months**										
No	180 (49)	48 (55)	51 (50)	81 (44)	78 (55)	42 (44)	60 (45)	82 (51)	48 (47)	50 (48)
Yes	190 (51)	37 (45)	51 (50)	102 (56)	63 (45)	53 (56)	74 (55)	80 (49)	55 (53)	74 (55)

*COVID-19, coronavirus disease 2019*.

A comparison by gender indicates that men had a greater rate of acceptance for the vaccine than women (57 vs. 44%, as a proportion within each category by gender), while women had a higher rate of refusal and undecided responses. Our test of means revealed that the differences in the response rates in the baseline scenario were statistically significant, with a Pearson's chi-squared (2) = 6.23; Pr = 0.044. Additionally, we did not find statistically significant differences by income, education, or health insurance system.

### Preference for a Hypothetical Vaccination Against Coronavirus Disease 2019 Under Three Vaccine Scenarios

**Figure 1:**
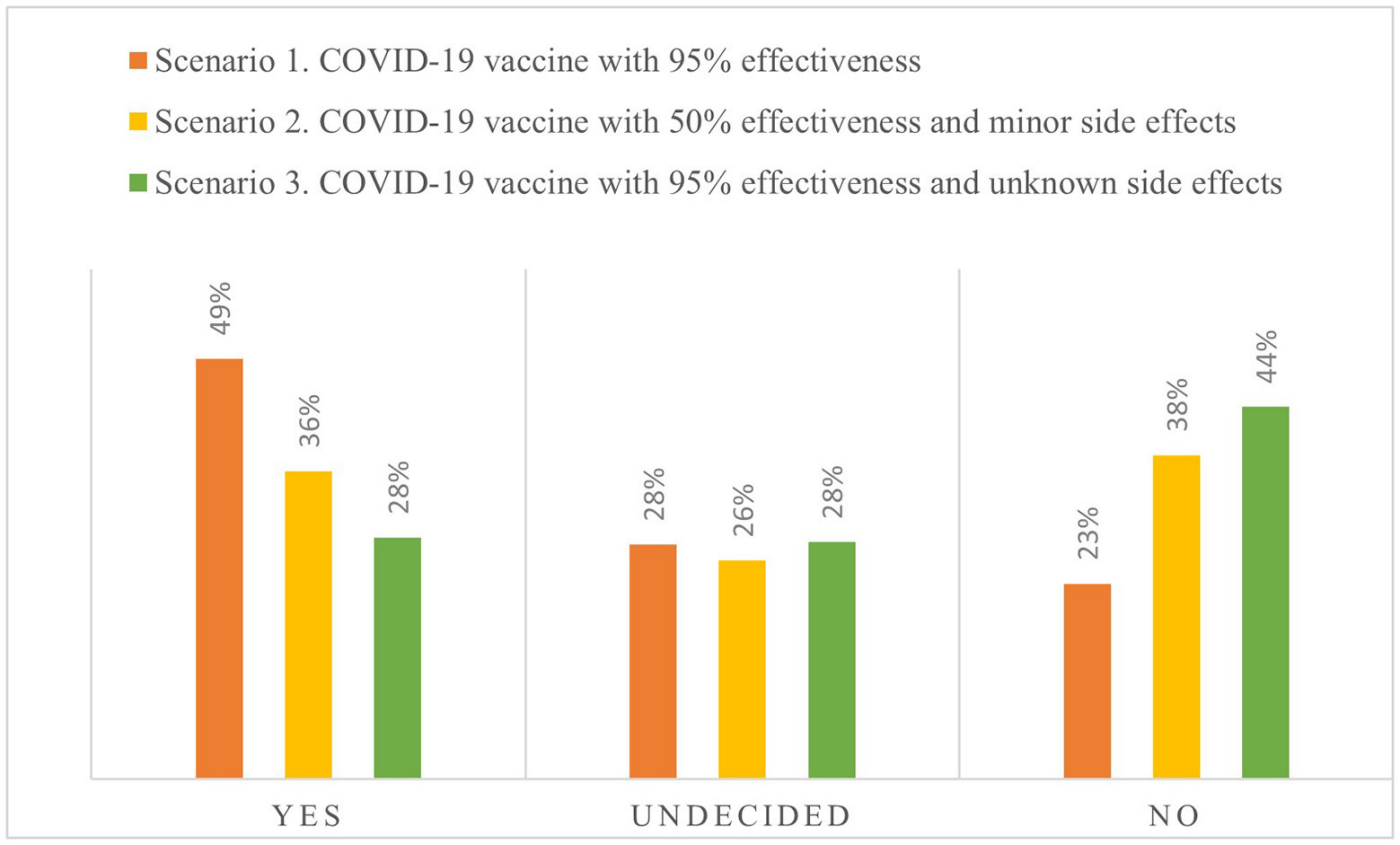
Preferences for a hypothetical vaccine against coronavirus disease 2019 (COVID-19) under three options.

We defined three scenarios to observe the hypothetical preference for a vaccine, the results of which are presented in Figure 1. In Scenario 1, we observed that ~49% of the respondents were willing to be vaccinated and 28% were undecided, indicating 77% were potentially willing to be vaccinated. These percentages change significantly if the side effects are unknown (Scenario 3), decreasing respondents' willingness to be vaccinated to 28% and increasing rejection from 23 to 44%. The percentage of undecided respondents was quite similar among the three scenarios.

Additionally, the age composition for acceptance, rejection, or indecision regarding the vaccine changed according to the vaccination scenarios (Table 1). Comparing both cases relative to the baseline revealed that indecision decreased in favor of rejection at 95% effectiveness but with unknown side effects, while indecision in favor of rejection decreased at 50% effectiveness but with minor side effects. Furthermore, the increase in the rejection rate was greater in Scenario 3 (57%) than in Scenario 2 (6%).

### Reasons Why Respondents Avoid Vaccination

**Figure 2:**
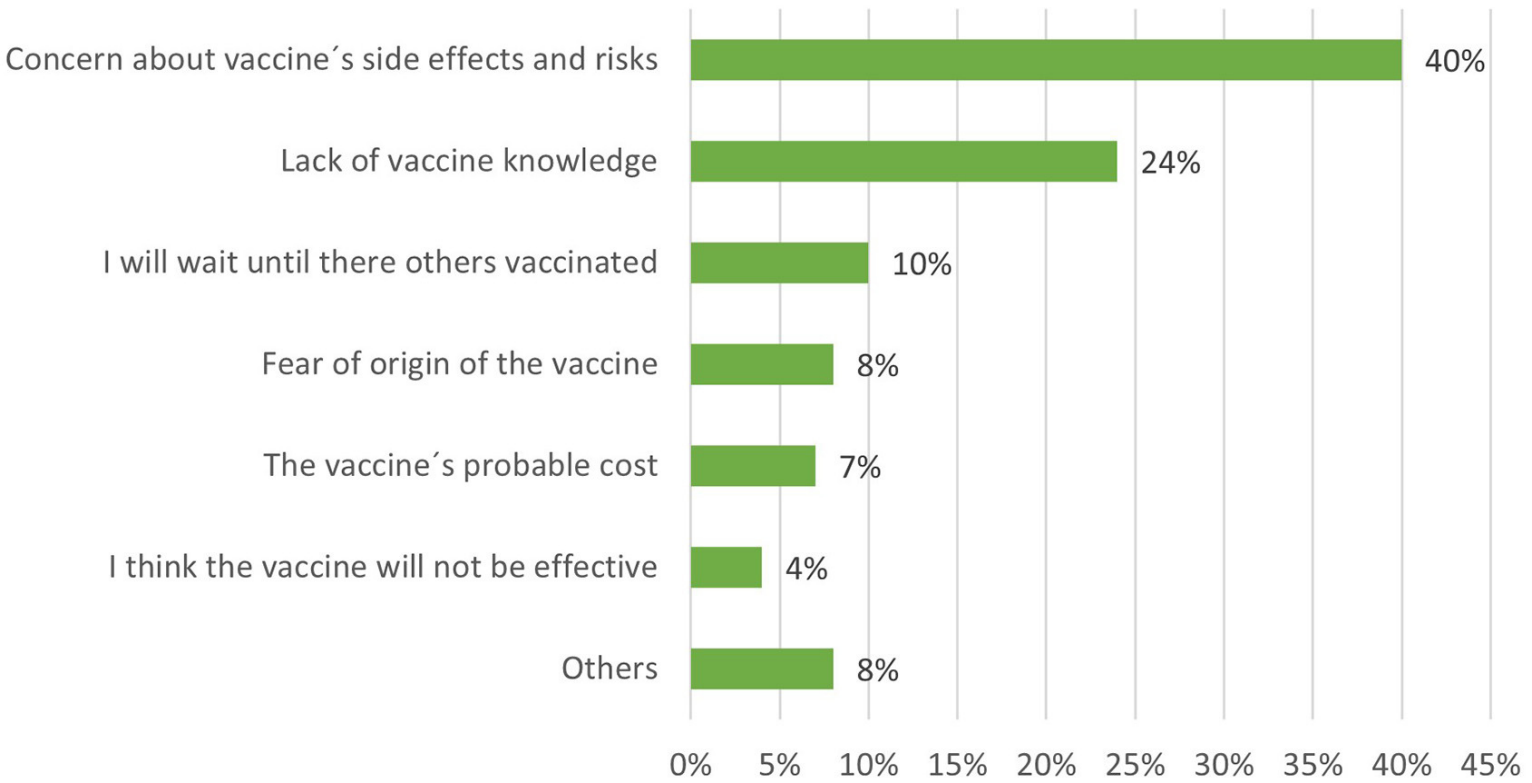
Reasons why respondents may avoid vaccination.

All those who responded to the survey were asked to mention the main reason that could lead them to avoid vaccination. The first-ranked reason was the vaccine's side effects and extent of risk, which is consistent with the information presented in the previous section. The second-ranked reason was the lack of knowledge of the vaccines, and the third-ranked reason was that they would prefer others to be vaccinated first (Figure 2).

Additionally, we show the refusal to vaccinate rate disaggregated by age range, gender, and education level (Table 2). The age category indicates that respondents between ages 30 and 49 considered their concerns with a vaccine's side effects and risks as the main reason for rejection. The other respondents distributed their preferences among all the alternatives, with percentages fewer than 10%. Similarly, the refusal rate by level of education shows that more educated people rejected the vaccine more often because of risks and side effects (graduated: 17%; university degree: 13%) than people with lower levels of education (high school: 2.7%). Likewise, those with a higher level of education showed a higher rejection rate due to a lack of knowledge of the vaccine (university degree 11%) than people who had a high school education (3%). Women rejected the vaccine more than men, mainly because of concern about side effects (women: 26.2% vs. men: 13.8%) and because of a lack of knowledge about the vaccine (women: 11.9% vs. men: 11.3%).

**Table 2:** Reasons why respondents may avoid vaccination by gender, age, and education (percentage of frequencies).

	**Concern about vaccine's side effect and risks**	**Lack of vaccine knowledge**	**I will wait until there others vaccinated**	**Fear of origin of the vaccine**	**The vaccine's probable cost**	**I think the vaccine will not be effective**	**Others**	**Total**
**Gender**								
Female	26.22	11.89	6.76	4.59	4.32	1.08	3.51	58.38
Male	13.78	11.35	3.24	3.24	2.43	2.70	3.78	40.54
Not defined	0.27	0.27	0.00	0.00	0.00	0.27	0.27	1.08
Total								100
**Age**								
18–29	5.68	2.97	1.89	0.27	0.00	0.27	1.35	12.16
30–39	12.97	4.05	2.16	1.08	1.62	0.81	1.08	23.78
40–49	10.00	5.41	2.16	0.81	1.89	0.81	1.08	22.16
50–59	6.76	6.49	1.35	1.89	1.62	1.62	1.89	21.62
60+	4.86	4.59	2.43	3.78	1.62	0.81	2.16	20.27
Total								100
**Education**								
High school	2.70	2.97	0.81	0.81	1.35	0.00	1.62	10.27
Technical	7.30	3.24	2.70	0.81	0.54	0.00	0.81	15.41
University degree	13.24	11.35	3.51	2.16	2.70	1.89	2.97	37.84
Graduate degree	17.03	5.95	2.97	4.05	2.16	2.16	2.16	36.49
Total								100

### Adapted Health Belief Model

Table 3 presents the main variables and their definition as included in the adapted HBM. The frequency statistics indicate that the main beliefs that led individuals to vaccinate were the perceived benefit of protecting themselves and their families (90% strongly agree or agree), the action cues regarding the responses of their families during the pandemic (85% strongly agree or agree), the severity of complications from contracting COVID-19 (71% strongly agree or agree), and the benefit associated with the fact that the vaccine would reduce the fear of getting infected (70% strongly agree or agree) in considering a potential immunity against the disease.

**Table 3:** Health belief adapted model variable definition and vaccination preference.

	**Strongly disagree/not probable**	**Disagree/somewhat improbable**	**Neither agreement nor disagree/neutral**	**Agree/somewhat probable**	**Strongly agree/very probable**
**Barrier 1. Social networks indicate that vaccinating is inconvenient**					
Total	142 (38)	68 (18)	110 (30)	28 (8)	22 (6)
Not	27 (32)	17 (20)	28 (33)	8 (9)	5 (6)
Undecided	34 (33)	19 (19)	38 (37)	6 (6)	5 (5)
Yes	81 (44)	32 (17)	44 (24)	14 (8)	12 (7)
**Barrier 2. I think the vaccine will be very risky**					
Total	12 (3)	20 (5)	107 (29)	113 (31)	118 (32)
Not	3 (4)	3 (4)	26 (31)	23 (27)	30 (35)
Undecided		3 (3)	31 (30)	31 (30)	37 (36)
Yes	9 (5)	14 (8)	50 (27)	59 (32)	51 (28)
**Severity 1. I consider the severity of complications from contracting COVID-19**					
Total	8 (2)	24 (6)	75 (20)	106 (29)	157 (42)
Not	4 (5)	9 (11)	23 (27)	21 (25)	28 (33)
Undecided	1 (1)	6 (6)	23 (23)	28 (27)	44 (43)
Yes	3 (2)	9 (5)	29 (16)	57 (31)	85 (46)
**Severity 2. I think the vaccine will be ineffective**					
Total	52 (14)	108 (29)	121 (33)	55 (15)	34 (9)
Not	4 (5)	19 (22)	23 (27)	24 (28)	15 (18)
Undecided	7 (7)	19 (22)	23 (27)	24 (28)	15 (18)
Yes	41 (22)	62 (34)	48 (26)	19 (10)	13 (7)
**Severity 3. I have concerns regarding the side effects**					
Total	22 (6)	37 (10)	112 (30)	93 (25)	106 (29)
Not	2 (2)	4 (5)	16 (19)	21 (25)	42 (49)
Undecided	1 (1)	4 (4)	29 (28)	37 (36)	31 (30)
Yes	19 (10)	29 (16)	67 (37)	35 (19)	33 (18)
**Motivation 1. Religious reasons**					
Total	229 (62)	60 (16)	67 (18)	7 (2)	7 (2)
Not	54 (64)	16 (19)	12 (14)	2 (2)	1 (1)
Undecided	60 (59)	17 (17)	24 (23)	1 (1)	
Yes	115 (63)	27 (15)	31 (17)	4 (2)	6 (3)
**Motivation 2. The disease was invented by politicians and the pharmaceutical industry**					
Total	212 (57)	54 (15)	70 (19)	18 (5)	16 (4)
Not	41 (48)	11 (13)	21 (25)	2 (2)	10 (12)
Undecided	57 (56)	19 (19)	19 (19)	4 (4)	3 (3)
Yes	114 (62)	24 (13)	30 (16)	12 (7)	3 (2)
**Benefit 1. I would protect myself and my family**					
Total	10 (3)	5 (1)	23 (6)	54 (15)	278 (75)
Not	8 (9)	5 (6)	15 (18)	17 (20)	40 (47)
Undecided	-	–	7 (7)	20 (20)	75 (73)
Yes	2 (1)	–	1 (1)	17 (9)	163 (89)
**Benefit 2. The vaccine will reduce my fear of contagion**					
Total	22 (6)	20 (5)	68 (18)	123 (33)	137 (37)
Not	14 (16)	11 (13)	28 (33)	21 (25)	11 (13)
Undecided	–	8 (8)	22 (22)	46 (45)	26 (25)
Yes	8 (4)	1 (0.5)	18 (10)	56 (31)	100 (55)
**Benefit 3. The available vaccine is effective**					
Total	14 (4)	12 (3)	127 (37)	103 (28)	104 (28)
Not	11 (13)	6 (7)	40 (47)	10 (12)	18 (21)
Undecided	2 (2)	3 (3)	50 (49)	32 (31)	15 (15)
Yes	1	3 (2)	47 (26)	61 (33)	71 (39)
**Benefit 4. The available vaccine is safe**					
Total	17 (5)	12 (3)	139 (37)	95 (26)	107 (29)
Not	12 (14)	6 (7)	39 (46)	8 (9)	20 (24)
Undecided	2 (2)	3 (3)	49 (48)	31 (30)	17 (17)
Yes	3 (2)	3 (2)	51 (28)	56 (31)	70 (38)
**Cue_to_action 1. I will wait for others to be vaccinated**					
Total	63 (17)	40 (11)	97 (26)	98 (26)	72 (19)
Not	9 (11)	11 (13)	25 (29)	16 (19)	24 (28)
Undecided	5 (5)	5 (5)	26 (25)	42 (41)	24 (24)
Yes	49 (27)	24 (13)	46 (25)	40 (22)	24 (13)
**Cue_to_action 2. A lack of vaccine knowledge**					
Total	69 (19)	38 (10)	68 (18)	98 (26)	97 (26)
Not	14 (16)	11 (13)	10 (12)	22 (26)	28 (33)
Undecided	6 (6)	5 (5)	18 (18)	39 (38)	34 (33)
Yes	49 (27)	22 (12)	40 (22)	37 (20)	35 (19)
**Cue_to_action 3. The government's communication in response**					
Total	78 (21)	85 (23)	112 (30)	80 (22)	15 (4)
Not	31 (36)	20 (24)	19 (22)	13 (15)	2 (2)
Undecided	19 (19)	18 (18)	41 (40)	19 (19)	5 (5)
Yes	28 (15)	47 (25)	52 (28)	48 (26)	8 (4)
**Cue_to_action 4. Family's response to the pandemic**					
Total	2 (2)	15 (4)	39 (11)	154 (42)	160 (43)
Not	2 (2)	6 (7)	12 (14)	33 (39)	32 (38)
Undecided	-	1 (1)	15 (15)	41 (40)	45 (44)
Yes		8 (4)	12 (7)	80 (44)	83 (45)
**Cue_to_action 5. The Medical College of Chile recommended the vaccine**					
	26 (7)	14 (4)	109 (29)	108 (29)	113 (31)
	12 (14)	8 (9)	36 (42)	17 (20)	12 (14)
	2 (2)	3 (3)	26 (25)	44 (43)	27 (26)
	12 (7)	3 (2)	47 (26)	47 (26)	74 (40)
**Cue_to_action 6. My doctor recommended the vaccine**					
Total	48 (13)	45 (12)	104 (28)	81 (21)	92 (24)
Not	11 (13)	11 (11)	24 (28)	17 (20)	22 (26)
Undecided	14 (14)	11 (11)	30 (29)	24 (24)	23 (23)
Yes	23 (13)	23 (13)	50 (27)	40 (22)	47 (26)
**Susceptibility 1. Family with the possibility of contracting COVID-19**					
Total	70 (19)	96 (26)	138 (37)	33 (9)	33 (9)
Not	20 (5)	22 (6)	29 (8)	8 (2)	6 (2)
Undecided	13 (4)	31 (9)	37 (10)	11 (3)	10 (3)
Yes	37 (10)	43 (12)	72 (20)	14 (4)	17 (5)
**Susceptibility 2. A family member has chronic diseases**					
	**No**	**Yes**			
Total	82 (22)	288 (78)			
Not	17 (20)	68 (80)			
Undecided	21 (21)	81 (79)			
Yes	44 (24)	139 (76)			
**Susceptibility 3. Family or relative with COVID-19**					
Total	291 (79)	79 (21)			
Not	76 (89)	9 (11)			
Undecided	73 (72)	29 (28)			
Yes	142 (78)	41 (22)			
**Susceptibility 4. Chile has one of the highest infection rates per 1,000 inhabitants**					
Total	92 (25)	278 (75)			
Not	24 (28)	61 (72)			
Undecided	34 (33)	68 (67)			
Yes	34 (19)	149 (81)			

*COVID-19, coronavirus disease 2019*.

Additionally, the results from the descriptive statistics are consistent with respondents' preferences for the scenarios, as the former demonstrate that people cared more about the potential risks from vaccination than its effectiveness. In other words, individuals perceived or preferred aspects associated with safety and fewer side effects over the vaccine's effectiveness. Specifically, the vaccine's health risks were a relevant barrier for a relatively high number of respondents (66% strongly agree or agree), while the perceived benefits from having an available, effective vaccine were slightly fewer (56% strongly agree or agree).

Another noteworthy aspect is the barrier associated with social media's potential negative influence on the decision to be vaccinated, where respondents significantly disagreed and strongly disagreed (46%); additionally, 30% were indifferent (Table 3). Regarding the frequency of responses by severity, the results indicate that the factors that could potentially influence the vaccination decision included whether the respondent had a family member with a chronic disease and the country's infection rate.

Table 4 displays the estimation results from the multinomial logit regression model, which indicate that the model containing the full set of predictors represents a significant improvement in fit relative to a null model (logit regression chi-squared *p* < 0.001); therefore, it can be inferred that at least one population slope is non-zero. Hausman's test demonstrated that the answers exist independent of other alternatives. According to McFadden's pseudo R-squared value, we can conclude that the full model containing our predictors represents a 37% improvement in fit relative to the model; and the mean of the variance inflation factor (VIF) was 1.92, indicating that there was no collinearity. Thus, the model had sufficient statistical validity.

**Table 4:** Multinomial logit estimations based on the Health Belief Model to get vaccination.

**Base outcome assigned to “I am willing to be vaccinated” (yes)**	**“I refuse to get vaccinated” (no)**				**“I have not yet decided whether to vaccinate” (hesitant)**			
**Variable^**+**^**	**RRR^++^**	**SE^+++^**	**Coef. ^++++^**	**SE^+++^**	**RRR^++^**	**SE^+++^**	**Coef. ^++++^**	**SE^+++^**
Susceptibility 1. Family with the possibility of contracting COVID-19	1.1786	0.2395	0.1642	0.2031	1.440**	0.2382	0.3650**	0.1653
Susceptibility 2. A family member has chronic diseases	2.8580**	1.4057	1.050**	0.4918	1.8781	0.7688	0.6302	0.4093
Susceptibility 3. Family or relative with COVID-19	0.1190***	0.0720	−2.129***	0.6053	0.9982	0.3754	−0.0018	0.3761
Susceptibility 4. Chile has one of the highest infection rates per 1,000 inhabitants	0.5942	0.2764	−0.5206	0.4652	0.4616**	0.1763	−0.7731**	0.3820
Severity 1. I consider the severity of complications from contracting COVID-19	0.6627**	0.1327	−0.4115**	0.2002	0.6873	0.1219	−0.3750**	0.1774
Severity 2. I think the vaccine will be ineffective	1.5400**	0.3085	0.4318**	0.2003	1.2255	0.2144	0.2034	0.1749
Severity 3. I have concerns regarding the side effects	2.3327***	0.5046	0.8470***	0.2162	1.6922***	0.3110	0.5261***	0.1838
Benefit 1. I would protect myself and my family	0.4846**	0.1531	−0.7243**	0.3158	0.8767	0.2649	−0.1316	0.3021
Benefit 2. The vaccine will reduce my fear of contagion	0.4993**	0.0952	−0.6945***	0.1961	0.5724***	0.1051	−0.5579***	0.1835
Benefit 3. The available vaccine is effective	0.4256*	0.1967	−0.8540*	0.4620	0.1801***	0.0760	−1.714***	0.4222
Benefit 4. The available vaccine is safe	2.1697*	0.9392	0.7745*	0.4328	2.253**	0.8936	0.8121**	0.3967
Barrier 1. Social networks indicate that vaccinating is inconvenient	0.8670	0.1833	−0.1428	0.2115	0.6030***	0.1143	−0.5059***	0.1896
Barrier 2. I think the vaccine will be very risky	1.5877**	0.3045	0.4623**	0.1917	1.5999***	0.2563	0.4699***	0.1602
Cue_to_action 1. I will wait for others to be vaccinated	1.1646	0.1996	0.1524	0.1713	1.5520***	0.2440	0.4389***	0.1573
Cue_to_action 2. A lack of vaccine knowledge	1.0858	0.1698	0.0823	0.1563	1.6910***	0.2393	0.5253***	0.1415
Cue_to_action 3. The government's communication in response	1.1573	0.3177	0.1461	0.2745	1.8963***	0.4710	0.6399***	0.2484
Cue_to_action 4. Family's response to the pandemic	0.6305**	0.1215	−0.4613**	0.1926	1.1651	0.1801	0.1528	0.1546
Cue_to_action 5. The Medical College of Chile recommended the vaccine	0.8019	0.1945	−0.2208	0.2424	1.2431	0.2716	0.2176	0.2185
Cue_to_action 6. My doctor recommended the vaccine	0.8007	0.1986	−0.2223	0.2479	1.2884	0.2816	0.2534	0.2186
Motivation 1. Religious reasons	0.4449**	0.1340	−0.8000**	0.3011	0.9382	0.2280	−0.0637	0.2430
Motivation 2. The disease was invented by politicians and the pharmaceutical industry	0.7539	0.1770	−0.2825	0.2347	1.1403	0.2437	0.1314	0.2137
Gender	0.4586**	0.1815	−0.7795**	0.3956	0.7495	0.2447	−0.2883	0.3265
Level of Income	1.4423**	0.2098	0.3662**	0.1454	1.2850**	0.1536	0.2508**	0.1195
Relative_work_health_sys	2.5477**	1.0298	0.9352**	0.4041	0.7383	0.2557	−0.3034	0.3464
Work_health_sys	1.150558	0.6585	0.1403	0.5723	0.2611**	0.1777	−1.342**	0.6806
Constant	70.68541	184.18	4.258	2.606	0.0044	0.0112	−5.421	2.536
Number of observations	370							
LR chi^2^(50)	284.8							
Prob > chi^2^	0.0000							
Log likelihood	242,888							
Pseudo R^2^	36.96							

*COVID-19, coronavirus disease 2019*.

*p < 0.10; **p < 0.05; ***p < 0.01.

*^+^Variables are as defined in Table 2*.

*^++^RRR, relative risk rate*.

*^+++^SE, standard error*.

*^++++^Coef., Coefficient*.

In estimating the model, we assigned the “Yes, I will be vaccinated” category as a baseline, with no coefficients or test provided in this category. Therefore, we interpreted the coefficients' values by comparing the baseline relative with the “No, I refuse to be vaccinated” and “I have not yet decided whether to vaccinate (undecided)” categories. Taking the “Yes, I will be vaccinated” response as a baseline is convenient, as this permits us to analyze the independent variables that significantly predict whether a respondent falls into the baseline or comparison category. In other words, we could then observe the variables that significantly predict whether a respondent was anti-vaccine or undecided instead of pro-vaccine. Subsequently, we could identify the independent variables relevant in creating potential public policies for these vaccinations.

### Determinants of the Probability of Hesitancy

We considered the coefficients of the multinomial logit estimate that were statistically significant to identify the positive and negative determinants of the probability of hesitancy (Table 4). On the one hand, the variables that reduced the logarithmic relative probability of hesitancy versus being vaccinated against COVID-19 were the increased availability of an effective vaccine (Coef.: −1.71; 99%), work in the health sector (Coef.: −1.34; 99%), the increase in the contagion rate per 1,000 inhabitants (Coef.: −0.77; 95%), the social network indicating that vaccinating is inconvenient and increased belief that the vaccine reduces fear of contagion (Coef.: −0.56; 99%), and the greater the perceptions of health complications generated by COVID-19 (Coef.: −0.38; 95%). On the other hand, the main variables that increased this relative probability were increased positive perceptions about the government's communication response to the pandemic (Coef.: 0.64; 99%), the greater fear of side effects (Coef.: 0.53; 95%) and the belief that the vaccine is risky (Coef.: 0.47; 99%), the increase in lack of general knowledge of the vaccine (Coef.: 0.53; 99%), a preference for waiting for others to get vaccinated first (Coef.: 0.44; 99%), and the level of income (Coef.: 0.25; 95%).

The expected risk of rejection was lower for individuals who had a greater belief in the severity of the complications of contracting COVID-19 (RRR: 0.662; 99%), those who think that the vaccine could protect themselves and their families (RRR: 0.484; 95%), the perception that the available vaccine is effective (RRR: 0.426; 90%), the better the family's response to the pandemic (RRR: 0.631; 95%), and men compared with women (RRR: 0.459; 95%). The probability of rejection compared with the group that would be vaccinated, measured in relative risk, increased mainly with concern about side effects (RRR: 2.33; 99%), the belief that the vaccine will not be effective (RRR: 1.54; 95%) or that it will be very risky (RRR: 1.59; 95%), and level of income (RRR: 1.44; 95%).

### Determinants of the Probability of Refusal

The variables that reduced the logarithmic relative probability of refusal versus being vaccinated against COVID-19, considering the estimation coefficients, were increase in family members who have contracted COVID-19 (Coef.: −2.13; 99%), increased availability of an effective vaccine (Coef.: −0.85; 90%), the perception that the vaccine could protect oneself and others (Coef.: −0.72; 95%), increased perceived benefits of the vaccine reducing fear of contagion (Coef.: −0.69; 99%), the family's improved response to the pandemic (Coef.: −0.46; 95%), and an increased perception regarding the severity of the infection caused by SARS-CoV-2 (Coef.: −0.41; 95%). The relative probability of refusal increases while the relative probability of rejection increases with the increase in concern about side effects (Coef.: 0.85; 99%) and risk (Coef.: 0.46; 95%), and the growth of the belief that the vaccine could be ineffective (Coef.: 0.43; 95%), among others that are presented in Table 4.

The RRRs indicate that if an individual increases the score in the items that are statistically significant in the model by one point, it would be expected that the relative risk of rejection of the vaccine will decrease in relation to its acceptance, since other variables in the model remain constant. The most relevant items that showed this behavior were consideration of the severity of the complications of becoming infected with SARS-CoV-2 (RRR: 0.67; 95%), the family's response to the pandemic (RRR: 0.63; 95%), the expected benefits of protecting oneself and others (RRR: 0.48; 95%), and family or relatives with COVID-19 (RRR: 0.12; 99%). Additionally, for women in comparison with men, the relative risk of rejection in relation to the acceptance of the vaccine would be expected to decrease by a factor of 0.46 (95%), since the other variables in the model remain constant.

## Discussion

Authorities worldwide have addressed the COVID-19 pandemic by promoting preventive measures based on hygiene and social distancing. As the disease continues to expand, nonetheless, it is expected that the next step in this battle involves developing and distributing a vaccine. However, individuals must be willing to be vaccinated to ensure widespread global immunity. In this regard, our results from sampling 370 Chileans revealed that 49% of respondents were willing to be vaccinated, with 28% undecided and 23% refusing vaccination altogether. Overall, these respondents would consider a hypothetical vaccine with 95% efficacy and minor side effects. Thus, we found that 77% of individuals would potentially be vaccinated. This is consistent with other recent findings, in the sense that the undecided group is a more flexible group and with appropriate interventions they are more likely to change from being undecided to acceptance of a vaccine (28).

In addition, the proportions by groups of acceptance, refusal, and hesitancy are similar to those obtained by Lazarus et al. (29) and Wong et al. (5), but lower than those of Harapan et al. (24) who found an acceptance rate of 93.3% for a vaccine with 95% effectiveness. Our work differs from previous studies in that we evaluated how the vaccine's acceptance changes given hypothetical variations in efficacy or side effects under three scenarios. In this regard, we found that more individuals exhibited higher rejection rates for a highly effective vaccine with unknown side effects (44%) than when faced with a less effective vaccine with lesser side effects (38%). This illustrates the importance of not only rigorous human testing of the vaccine but also communicating the vaccine's side effects to society, as this will directly affect individuals' preferences and their vaccination decisions. It should be noted that this contradicts what has been stated in some studies; for example, Dubé et al. (30) indicated that the information on effectiveness and side effects did not affect the people's decision about getting vaccinated.

Although we identified the determinants of hesitation or refusal compared with a group of individuals who were willing to be vaccinated, our study also provides other findings similar to those of Wong et al. (5). Both studies demonstrated that decreasing the fear or concern of getting the illness was a key aspect in determining the vaccination decision; further, this vaccine would help to reduce the possibility of contagion. However, our model exhibits a better goodness of fit and more statistically significant variables that explain the indecision toward or rejection of the vaccine, compared with the one developed by Wong et al. (5). We found that other key belief-related variables that affect the decision not to vaccinate and/or indecision are complications from a SARS-CoV-2 infection; an effective vaccine's availability; fear of the vaccine's side effects and health risks; the disease's prevalence, or rate per 1,000 inhabitants; the roles of social media and government authorities; and the recommendations from health or medical unions. All these variables were statistically significant, with important implications in designing vaccination campaigns. As previous literature has yet to consider three of our variables, our model reduces potential biases due to omitted relevant variables by considering Mokhtarian's (23) work.

The literature on the HBM and vaccines does not address elements associated with altruism as a motivating or benefit variable. However, we included it in the model (benefit 1), and we found that this was a relevant aspect, given the statistically significant finding (Table 4). Thus, the probability of rejection of the vaccine was reduced by the variable that measured altruistic motivation. Thus, people would be vaccinated to protect not only themselves but also their loved ones; in other words, there could be less rejection of the vaccine if individuals believe that it helps reduce the transmission of COVID-19. This is consistent with the experiment conducted by Rieger (31), who found that both selfish and altruistic motivations were effective in convincing people to get vaccinated. In addition, Rieger proposed that social preferences affect health behaviors that impact others (32). Thus, this potential benefit of vaccination (protecting others) can be used as a promotional element for the vaccination campaign. According to Farboodi et al. (33), knowing the social impact of individual behavior can be a tool for the formulation of public health policies.

We found that a lower probability of refusal and being undecided manifested in individuals with relatives who contracted COVID-19 and the growth rate of infection per 1,000 in Chile. Therefore, susceptibility does affect individual preferences for the vaccine, which is consistent with Costa's (19) results. Additionally, young people had greater rates of rejection and hesitancy regarding vaccination. Consequently, communication strategies could be implemented to promote vaccination among young people as the main target group, as well as people who already had COVID-19 or with family members who had it, considering that the possibility of reinfection exists according to Centers for Disease Control and Prevention (34).

The identification of variables is key in formulating public health policies, as the HBM indicates that changes in an individual's behavior could be generated through the orientation or direction of barriers, benefits, severity, and susceptibility, among other factors (6). Furthermore, these changes could guide people toward objective behaviors that guarantee that a larger proportion of the population is vaccinated as a preventive measure. In other words, the variables that we discovered are those that should have the greatest influence through communication campaigns that promote the COVID-19 vaccination.

As indicated by results from the H1N1 vaccination campaign in Indiana as examined by Jones et al. (6), an inadequate communication approach was used because the campaign focused on only two elements: severity and susceptibility. In contrast, Fournet et al. (35) found that concern about side effects was a relevant aspect to explain anti-vaccine movements in Europe. Therefore, the design of health campaigns for the COVID-19 vaccine must consider all aspects and not focus only on one. The aspects to be considered are related to the beliefs of individuals that would have the effect of reducing the probability of rejection or hesitancy. Our results demonstrate that there are many variables associated with vaccine-related actions or cue to action, severity (side effects and effectiveness), benefits, barriers, and motivations that are relevant to individuals' decision making.

Social networks' influence was statistically significant as an explanation for the probability of indecision. Specifically, this result indicates the risk of vaccination strategies as generated by online communities, which can encourage the dissemination of false, biased, or inaccurate information. According to Arfini et al. (36) and Roozenbeek et al. (37), social media are diffusers of ignorance and are exploited by anti-vaccine movements; however, this misinformation is based on health risks as well as conspiracy theories. Consequently, the variable regarding the potential belief that “the COVID-19 disease is a political or pharmaceutical invention” was not statistically significant in our model.

We also found that the government's communication response would affect the probability of vaccination, consistent with an Australian case of non-compliance with COVID-19 measures associated with government confidence (38). Ward et al. (9, 39) indicate that trust in the authorities contributed to the adoption of the vaccine. However, the findings differ slightly from Clark et al. (40), who showed that trust in the government had a low influence on individual decisions to take other preventive measures against COVID-19 (including mask wearing, social distancing, handwashing, and staying at home). This could be due to the fact that preventive measures are valued differently by people, such as, valuing the vaccine more than the use of a mask, which could be explored in future research.

Considering the importance of social media and trust in the government, communication from the government is considered key to promote vaccination as a measure to prevent contagion. Further, it highlights the need for health authorities to use scientific data to counteract the erroneous information disseminated on social media and adequately inform citizens of the COVID-19 vaccine's benefits and risks. There are studies that indicate the need to incorporate the relationship between the information transmitted by governments and the role of social networks in the design of vaccination campaigns against COVID-19 (11, 41). Thus, people's trust can be fostered through clear, transparent, and timely information based on scientific knowledge. According to Bles et al. (42), such information would be perceived as more open and transparent and therefore result in a greater willingness of people to get vaccinated (37), which may be achieved by following the recommendations of Mheidly and Fares (43). Similarly, our statistically significant variables mentioned in the *Results* section can help authorities to design communication strategies focused on anti-vaccine movements, as such variables that can help them understand the beliefs of those who reject vaccines. In this regard, we observe that religious beliefs statistically explained the probability of vaccine rejection, which is typically one variable that influences anti-vaccine decisions (44).

### Strengths and Limitations

There are several strengths and limitations of our study that deserve mentioning. Among the strengths are identification of the variables that affect both the probability of refusal and hesitancy of being vaccinated for COVID-19 in the context of the HBM. Among these variables, the role of social networks, altruism, the perception of severity of the disease, fear of side effects, and susceptibility to contagion are prominent. These can guide the design of vaccination campaigns targeting messages to undecided or anti-vaccine groups, such as young people. Another notable strength is the use of three possible scenarios to be able to determine the intention to get vaccinated, showing that individuals prefer having fewer side effects more than the effectiveness of the vaccine itself. Furthermore, the scenario with the least hesitation was one in which the vaccines were approved in both the United States and Europe.

With regard to limitations, our sample includes a high proportion of people with relatively high education levels, and a convenience sampling and snowball recruitment method was used. This limits the generalizability of the results. Another limitation is that the results of probability of acceptance, refusal, and hesitancy are marked by the temporal context of the pandemic; therefore, they could change over time.

## Conclusions

The analysis of preferences for different hypothetical vaccines indicates that people value a vaccine's minor side effects more than its effectiveness. This provides evidence regarding the importance of rigorous human testing for any vaccine, and the significance of communication with society regarding its side effects. Collectively, these will directly affect individuals' vaccination preferences and decisions.

We also revealed the key health beliefs that positively or negatively affect the refusal and hesitancy of a hypothetical COVID-19 vaccine. These should be used in formulating public health policies, and specifically in designing promotional strategies for the vaccine. Furthermore, specific promotional campaigns can be aimed toward different anti-vaccine and undecided groups, such as, younger people, influencing beliefs, cue to action, perception of severity (side effects and effectiveness), benefits, barriers, and motivations.

On the one hand, the variables that explain rejection could be used to counter anti-vaccine movements through public health communication strategies. These strategies should effectively address citizens' concerns with side effects and potential health risks by disseminating information through not only associations with doctors and health personnel but also social networks. On the other hand, the promotional strategy to mitigate hesitation could focus more on the government's communication response and increasing the population's knowledge of the vaccine, in addition to its risk factors, effectiveness, and side effects. However, even hesitant groups could be protected through herd immunity given sufficiently high vaccination rates in the general population. This could boost the vaccination rate as a result, which is key to controlling COVID-19 outbreaks and recurring infections.

## Data Availability Statement

The raw data supporting the conclusions of this article will be made available by the authors, without undue reservation.

## Ethics Statement

Ethical review and approval was not required for the study on human participants in accordance with the local legislation and institutional requirements. The patients/participants provided their written informed consent to participate in this study.

## Author Contributions

The authors contributed equally to the writing of this manuscript.

## Conflict of Interest

The authors declare that the research was conducted in the absence of any commercial or financial relationships that could be construed as a potential conflict of interest.
